# Association of Sepsis Mortality with Specific Cancer Sites and Treatment Type: The Multiethnic Cohort Study

**DOI:** 10.3390/jpm11020146

**Published:** 2021-02-19

**Authors:** Yurii B. Shvetsov, Mari H. Ogino, Natalija Glibetic, Chloe B. Asato, Lynne R. Wilkens, Loïc Le Marchand, Michelle L. Matter

**Affiliations:** Cancer Center, University of Hawaii, Honolulu, HI 96813, USA; YShvetso@cc.hawaii.edu (Y.B.S.); moginosp@gmail.com (M.H.O.); glibetic@hawaii.edu (N.G.); asato9@hawaii.edu (C.B.A.); Lynne@cc.hawaii.edu (L.R.W.); Loic@cc.hawaii.edu (L.L.M.)

**Keywords:** cancer, sepsis, cancer treatment, mortality

## Abstract

Sepsis is a severe dysregulated immune response to infection. Sepsis deaths represent 9% of cancer deaths in the U.S. Evidence of the effect of specific cancer sites on sepsis mortality risk remains limited, and no research has evaluated the effect of cancer treatment on the risk of sepsis death. We examined whether cancer sites and treatments differentially affect the risk of sepsis death compared to other-cause mortality, among the 94,784 Hawaii participants in the Multiethnic Cohort, including 29,255 cancer cases, using competing risk Cox proportional hazards regression. Cancer diagnosis at any site was associated with similar increases in sepsis and non-sepsis mortality risk (HR: 3.39 and 3.51, resp.). Colorectal cancer differentially affected the risk of sepsis and non-sepsis mortality with a 40% higher effect on the risk of sepsis death compared with non-sepsis mortality (RRR: 1.40; 95% CI: 1.14–1.72). Lung cancer was associated with a significantly lower increase in sepsis compared to non-sepsis mortality (HR: 1.22 and 3.0, resp.; RRR: 0.39). Radiation therapy had no effect on sepsis mortality but was associated with higher risk of non-sepsis mortality (HR: 0.90 and 1.16, resp.; RRR: 0.76), whereas chemotherapy was associated with higher risk of both sepsis and non-sepsis mortality (HR: 1.31 and 1.21, resp.). We conclude that the risk of sepsis-related mortality is differentially affected by cancer sites and treatments. These associations were consistent across sexes and ethnic groups.

## 1. Introduction

Sepsis is defined as life-threatening organ dysfunction due to a dysregulated host response to infection and is associated with a death rate as high as 70% [1,2]. With an estimated 48.9 million cases of sepsis globally and more than 11 million recorded deaths in 2017 alone, sepsis is the leading cause of critical illness and death worldwide [3,4]. Several risk factors have been implicated in the etiology and progression of sepsis. Age, gender, presence of chronic health conditions, socioeconomic status, environmental factors and race/ethnicity have all been linked to the development and outcome of sepsis [5]. Currently, treatments remain supportive in the shape of antibiotics, vasopressors and fluid resuscitation. Cancer is one of the most frequent comorbidities in patients that develop sepsis, and cancer patients have higher hospital mortality rates than cancer-free patients [6,7]. In the United States cancer-associated sepsis is responsible for 9% of all cancer-related deaths annually [6]. While most reports of sepsis have been focused on the overall population, recently Abou Dagher and colleagues performed a retrospective chart study comparing the incidence of sepsis-related mortality between cancer and non-cancer patients. They reported that even with aggressive care, patients presenting with cancer-associated sepsis had a 2.3 times increase of hospital mortality compared with non-cancer septic patients [6]. Angus et al. [8] also found that patients presenting with cancer-associated sepsis have a nearly 37% mortality. Moreover, patients diagnosed with brain cancer, lung cancer, or hematological malignancies have been found at higher risk of developing sepsis [7,9].

Analysis of sepsis mortality data is complicated by the fact that sepsis typically accompanies other health conditions and is often coded as a contributing rather than primary cause of death (COD) [10]. While analyses of mortality due to cancer, cardiovascular disease (CVD) or other common causes usually rely on the primary COD only, the exclusive use of primary COD for sepsis may miss a high proportion of sepsis-related death events, which would underestimate sepsis mortality rates and bias comparisons between groups of patients (age groups, races/ethnicities, etc.). Any analysis of sepsis mortality risk should also take into account the effect of the factors of interest on all-cause mortality. A significant association between a risk factor and all-cause mortality may also manifest in the analysis of sepsis mortality, yet such an association will not be specific to sepsis. Thus, in sepsis mortality analyses that do not consider all-cause mortality, one cannot be certain whether the observed associations are specific to sepsis.

We have previously examined the differences and risk factors for sepsis mortality in the Multiethnic Cohort (MEC), considering deaths with sepsis as a primary COD [11]. In particular, we observed a 2-fold increase in mortality from cancer-associated sepsis in Native Hawaiians compared to non-cancer patients. In the present analysis, we sought to validate our prior results with an expanded definition of sepsis-related death, using the subsequently acquired information on contributing CODs in the Hawaii component of the MEC, and to determine which of the observed associations are specific to sepsis. Additionally, among cancer patients within the Hawaii MEC, we sought to examine the differential effect of cancer at specific sites and of cancer treatment on sepsis-related mortality.

## 2. Materials and Methods

### 2.1. Study Population

The MEC is a prospective cohort study designed to investigate the association of dietary, lifestyle, and genetic factors with the incidence of cancer among participants recruited from a multiethnic population. The study design and implementation have been described previously [12]. Briefly, between 1993–1996, over 215,000 men and women from five major ethnic groups (Whites, Japanese Americans, Latinos, African Americans, and Native Hawaiians), aged 45–75 years at recruitment and living in Hawaii or the Los Angeles, California area were identified through driver’s license files, voter registration lists and Medicare files and enrolled in the study. At entry, participants completed a 26-page self-administered baseline questionnaire, which contained questions on demographics, anthropometric measures, medical history, reproductive history (for women), diet and physical activity. The study protocol was approved by the Institutional Review Boards of the University of Hawaii and the University of Southern California.

### 2.2. Case Ascertainment

Incident invasive cancer cases were identified through linkage of the cohort to the Hawaii and California Cancer Registries and the Cancer Surveillance Program for Los Angeles County. Deaths were identified using state death files and the National Death Index. Deaths from sepsis were identified and classified as International Classification of Diseases, Ninth Revision (ICD-9) codes 038 and 995, or ICD-10 codes A40-A41. Cancer deaths were identified using the ICD-9 codes 140-208 or ICD-10 codes C00-C97 [13,14]. All-cause mortality included all deaths from other causes, including accidents and suicides. The Hawaii Department of Health state death files contained up to 20 causes of death for each record. All available causes of death (COD) were used to identify cancer and sepsis deaths. As no multiple COD data was available at the time of this analysis for the California part of the MEC, only the Hawaii part of the MEC was used in the present analysis. All death files were current as of December 31, 2015. Participants with no recorded death as of this date were censored.

### 2.3. Statistical Analysis

Analyses were limited to 45,314 male and 49,470 female Hawaii participants who identified with one of the five main MEC ethnic groups (white, African-American, Japanese-American, Native Hawaiian, Latino) and had no prior history of cancer at baseline. Separate counts were obtained for deaths with sepsis coded as the primary COD and with sepsis as a contributing COD, among all participants and among cancer cases only. The proportion of sepsis-related deaths among all deaths was calculated by sex and race/ethnicity.

The association of mortality risk with baseline demographic, behavioral and medical factors and with cancer diagnosis and treatment was modeled using Cox proportional hazards regression with age (years) as the time metric. Participants alive at end of follow-up (31 December 2015) were censored. Sepsis mortality, with sepsis coded as either primary or contributing COD, was the outcome of interest. An observed association between a risk factor and sepsis mortality could be due to an association with all-cause mortality and thus not specifically sepsis-related. To distinguish risk factors specifically associated with sepsis mortality, we used a competing risk model, which simultaneously modeled two outcomes, sepsis mortality and other-cause (non-sepsis) mortality, through data augmentation [15,16]. Hazard ratios (HR) and 95% confidence intervals (CI) were estimated for each of the two outcomes. Relative risk ratios (RRR), a measure of the difference in the association with sepsis and non-sepsis mortality, and 95% CIs were estimated using estimable contrasts across all categories of a risk factor.

Models were fit for all participants and for cancer cases only, and adjusted for sex and race/ethnicity. Baseline risk factors of interest were selected as those found significantly associated with primary-cause sepsis mortality in our prior analysis [11] and included body mass index (BMI; < 22 kg/m^2^, 22–24.9 kg/m^2^, 25–29.9 kg/m^2^, ≥ 30 kg/m^2^), education (12 y or less, 13 to15 y, ≥ 16 y), smoking (never smoker, past smoker, current smoker), alcohol consumption (none, < 14 g/d, ≥ 14 g/d), moderate-to-vigorous physical activity (< 2.5 h/week, ≥ 2.5 h/week), history of diabetes, heart disease, hypertension or stroke (yes, no). Missing values were modeled with a missing values category. In models for all participants, follow-up was defined from study entry to death or end of follow-up. Cancer status was modeled as a time-dependent variable. All baseline risk factors and cancer status were modeled simultaneously using a multivariable model. In models among cancer cases only, follow-up was defined from the date of cancer diagnosis to death or end of follow-up. Cancer at each common cancer site (colorectal, lung, breast, prostate) with cancer at all other sites as a reference, as well as cancer treatment (chemotherapy and radiation, yes/no) were modeled among cancer cases only and adjusted for all baseline factors. Separate models were fit for men and women, with all ethnicities combined and separately by ethnicity. The proportional hazard assumption for Cox models was verified by plotting scaled Schoenfeld residuals against time to event [17]. All analyses were conducted with SAS version 9.4 (SAS Institute, Inc., Cary, NC, USA). All P-values were two-sided, and *P* < 0.05 was defined as significant.

## 3. Results

### 3.1. Sample Characteristics and Mortality Summary

The 45,314 male and 49,470 female Hawaii MEC participants included in the analyses were followed for an average of 18.7 years (Table 1). Japanese Americans represented the largest racial/ethnic group, comprising over 43% of both men and women, followed by Whites (35.1% men and 30.6% women) and Native Hawaiians (13.0% men and 14.8% women). Participants of other ethnicity comprised about 10% of participants. The average age at cohort entry was 59 years. At baseline, 15.8% men and 14.6% women were obese, while 17.1% men and 13.9% women were current tobacco smokers. Over a third of participants had a history of high blood pressure.

Among the 94,784 participants followed for an average of 18.7 years, 37,386 deaths were recorded, of which 16,218 were from cancer and 1818 from sepsis (Table 2). A vast majority of sepsis-related deaths (75%) had sepsis coded as a contributing COD, rather than the primary COD. Cancer-associated sepsis mortality accounted for 4.7% (766) of cancer patient deaths and 4.9% of total deaths. For both cancer-associated and non-cancer mortality, the proportion of sepsis-related death was similar among men and women. Further examination of racial/ethnic groups demonstrated that Native Hawaiians experienced the highest proportion of sepsis-related mortality with 6.3% of all deaths and 5.8% of cancer deaths. Notably, Whites experienced the lowest proportion of sepsis-related mortality with 3.7% of all deaths and 3.8% of cancer deaths

### 3.2. Association of Sepsis Mortality with Baseline Factors

After adjustment for other predictors, the risk of both sepsis and other-cause mortality was statistically significantly elevated among participants who were obese (hazard ratio HR: 1.93 and 1.30 for sepsis and other-cause mortality, resp.), current smokers (HR: 2.21 and 2.42, resp.) or had a baseline history of comorbidities: diabetes (HR: 2.11 and 1.86, resp.), heart disease (HR: 1.34 and 1.57, resp.), hypertension (HR: 1.31 and 1.26, resp.), and stroke (HR: 1.59 and 1.64, resp.; Table 3). Cancer diagnosed during follow-up was also associated with higher risk of both sepsis and other-cause mortality (HR: 3.39 and 3.51, resp.). On the other hand, having a college or higher degree was associated with lower risk of both sepsis and other-cause mortality (HR: 0.70 and 0.77, resp.). These associations were consistent across sexes and racial/ethnic groups (Supplementary Table S1). Comparing the effect of these factors on sepsis and other-cause mortality risk, we observed that the association with obesity was significantly higher for sepsis mortality than for other causes of death (RRR: 1.48; 95% CI: 1.03–2.11). Conversely, the association of current smoking with sepsis mortality was significantly lower than with other-case mortality (RRR: 0.79; 95% CI: 0.63–0.99).

Among cancer cases, higher sepsis and other-cause mortality risk was associated with obesity at baseline (HR: 1.43 and 1.22, resp.), current smoking (HR: 1.63 and 2.33, resp.) and history of diabetes (HR: 1.58 and 1.41, resp.; Table 4). Among these factors, only current smoking differentially affected the risk of sepsis and other-cause mortality (RRR: 0.48; 95% CI: 0.33–0.69). Other non-cancer comorbidities were not associated with significantly higher risk of sepsis death.

### 3.3. Association of Sepsis Mortality with Cancer Sites

**Figure 1:**
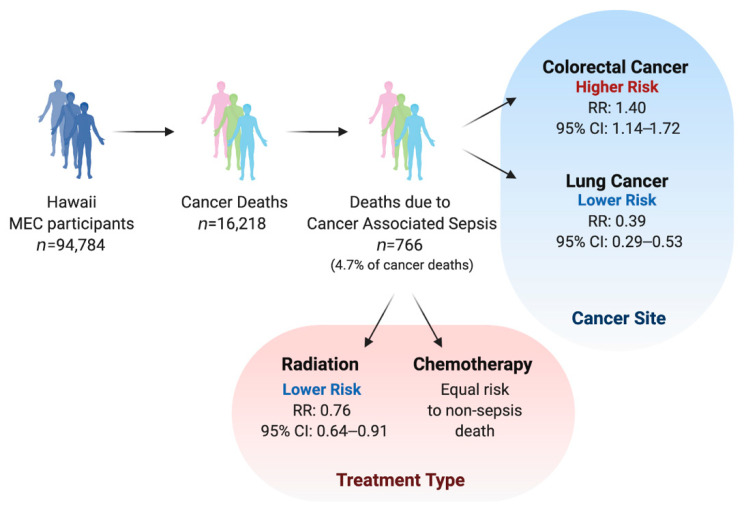
Summary of sample size and main findings.

We next investigated whether specific cancer sites increased cancer-associated sepsis mortality independently of the effect on overall mortality, in comparison to cancer at other sites (Figure 1). Colorectal cancer diagnosis was associated with a 40% higher effect on the risk of sepsis death compared with non-sepsis mortality (RRR: 1.40; 95% CI: 1.14–1.72; Table 4). Notably, this differential effect on sepsis mortality risk is due to a lower non-sepsis mortality (HR: 0.79) but no significant effect on sepsis mortality, compared to cancer at other sites. Lung cancer diagnosis was associated with a 61% lower effect on the risk of sepsis mortality than non-sepsis mortality when compared to all other cancer sites (RRR: 0.39; 95% CI: 1.14–1.72). This difference was due to significantly higher non-sepsis mortality risk (HR: 3.00) associated with lung cancer and higher (but to a lesser degree and non-significant) risk of sepsis mortality (HR: 1.22), compared to cancer at other sites.

### 3.4. Association of Sepsis Mortality with Cancer Treatment

Examining the association of cancer treatments, specifically chemotherapy and radiation, with sepsis and non-sepsis mortality yielded a surprising finding that radiation treatment differentially affected the risk of sepsis death, resulting in a smaller proportion of sepsis deaths among patients undergoing radiation treatment, compared to those who receive no radiation (RRR: 0.76; 95% CI: 0.64–0.91; Table 4). This difference was due to significantly elevated non-sepsis mortality risk (HR: 1.16) and non-significantly lower risk of sepsis mortality associated with radiation treatment. Chemotherapy was significantly associated with higher risk of both sepsis and non-sepsis mortality (HR: 1.31 and 1.21, resp.). While the effect on sepsis mortality was higher (RRR: 1.12), this difference was not statistically significant. These associations and differences were consistent across sexes and racial/ethnic groups (Table 4; Supplementary Table S2).

## 4. Discussion

In the present analysis we identified a number of risk factors associated with sepsis mortality. Further, we found that a number of these factors, in particular obesity and tobacco smoking, affect the risk of sepsis mortality differently than the risk of mortality due to other causes, and thus these associations can be considered specific to sepsis. Other factors, such as education and baseline comorbidities, affect other-cause mortality similarly to sepsis mortality and thus are not sepsis-specific. Of note, the latter also includes cancer diagnosis in comparison to non-cancer patients. While cancer diagnosis at any organ site was associated with higher risk of both sepsis and non-sepsis mortality, it did not differentially affect the risk of sepsis-related death.

A number of prior studies have examined the interplay between sepsis and cancer. Angus et al. [18] found that one out of six patients presenting with sepsis had an underlying malignancy. Williams and colleagues [7] determined that patients with cancer-associated sepsis presented with increased comorbid conditions, increased mortality and were older compared to cancer patients without sepsis. This study did not examine cancer-associated septic patients in comparison with non-cancer septic patients. Abou Dagher et al. examined cancer-associated sepsis patients vs. non-cancer septic patients and reported that patients with cancer-associated sepsis present with decreased comorbid conditions and are younger compare to the general population [6]. Liu et al. examined specific cancer sites and found that sepsis is significantly associated with 9 cancer types including thyroid, prostate, colon, rectum, lung, and liver and follicular lymphoma and melanoma [19].

Our results agree with previous reports of higher risk of sepsis mortality for cancer patients but suggest that this association extends from that for all-cause mortality and is not specific to sepsis. The non-significantly higher risk of sepsis death associated with colorectal cancer in our study (HR: 1.06) was similar to that reported by Liu et al. [19] (adjusted odds ratio, aOR: 1.12-1.13); however, coupled with the observed lower risk of other-cause mortality for colorectal cancer patients in our study, compared to cancer at other sites, this results in a much stronger differential effect. We also found a non-significantly higher risk of sepsis mortality among lung cancer patients (HR: 1.22), which was again in agreement with the results of Liu et al. [19] (aOR: 1.17); however, this risk increase was much smaller than that for other-cause mortality, suggesting a smaller proportion of sepsis deaths among lung cancer patients compared to cancers at other sites.

Although there was some variability in the risk estimates across racial/ethnic groups, the differential effect on sepsis mortality observed for the entire study population was exhibited in all three racial/ethnic groups, suggesting that the observed differences are not limited to particular races/ethnicities. We did, however, observe a higher proportion of sepsis deaths among Native Hawaiians, which is in agreement with our prior results based on primary COD [11]. While this finding may potentially be explained by higher prevalence of risk factors (e.g., obesity) among Native Hawaiians, we do not discount the possibility that genetic factors may also be at play. Genetically inherited risk factors impact infection rates and may in part contribute to racial/ethnic differences in sepsis mortality [18,20].

Cancer treatment options may also impact cancer-associated sepsis mortality risk. This is the first study to examine the association of cancer treatment type with sepsis and other-cause death risk. In our analysis radiation treatment was associated with higher risk of non-sepsis death but did not affect sepsis mortality, suggesting a smaller proportion of sepsis deaths among radiation therapy patients. Our findings point to a role for radiation treatment in cancer patients more susceptible to sepsis. It may be that radiation is less detrimental to the immune system and microbiome, both of which have been implicated in sepsis risk [21,22,23]. Chemotherapy was associated with higher risk of sepsis mortality in our study, and this effect was non-significantly higher than the increased risk of other-cause mortality. Cancer patients have a suppressed immune system and this suppression may be increased by chemotherapy, thereby enhancing cancer patient susceptibility to sepsis [16]. This differential effect of cancer treatment types on sepsis vs. other-cause mortality may help inform clinical decisions in treating cancer patients. For example, more aggressive infection prevention and screening measures could be utilized for patients undergoing chemotherapy, compared to those undergoing radiation treatment. Similarly, ethnic populations more prone to cancer-associated sepsis, such as Native Hawaiians, could benefit from increased clinical observation and early detection of sepsis, in addition to more aggressive treatment upon a sepsis diagnosis.

The strengths of this study include its large sample size, long follow-up period and multiethnic composition, which allowed us to assess sepsis mortality risk over long (>15 y) periods of time and across sex/racial/ethnic groups. By considering death with sepsis as a contributing COD, we were able to quadruple our event count. We considered cancer diagnosis as a time-dependent factor, which allowed us to properly account for its effect only after the date of diagnosis. Finally, by simultaneously modeling other-cause mortality risk, we were able to identify factors that differentially affect sepsis mortality, compared to other-cause mortality. Among limitations of the present analysis, we note, first, our inability to account for sepsis diagnoses which may not have necessarily led to death. Information on such diagnoses was not available in our study. Second, the relatively low rate of sepsis mortality among whites may limit the clinical utility of our results for this racial/ethnic group. Lastly, this and other studies of sepsis are affected by the lack of a clear definition of sepsis and inconsistencies in coding causes of death and death certification [10,24]. Future research would benefit from proposed changes to the sepsis definition and coding practices in the clinic [25,26,27].

## 5. Conclusions

This study is one of a few to examine sepsis mortality risk and specific cancer sites. It is the first study to evaluate cancer treatment options and the risk of cancer-associated sepsis. We found that colorectal and lung cancer, as well as radiation treatment, affect the risk of sepsis-related mortality differently than the risk of other-cause mortality. Chemotherapy increases the risk of sepsis mortality in line with an increase in other-cause mortality, whereas radiation treatment does not. Further understanding and identification of factors affecting the incidence and prognosis of sepsis may aid the development of effective treatment approaches for patients susceptible to sepsis.

## Figures and Tables

**Table 1:** Study sample characteristics, Multiethnic Cohort (Hawaii part).

	Men	Women
Baseline characteristics	*N* = 45,314	*N* = 49,470
Age at cohort entry (years) ^a^	59.3 ± 9.2	59.0 ± 9.1
Mean follow-up (person-years) ^a^	18.0 ± 5.8	19.3 ± 4.7
Total follow-up (person-years)	814,432	954,829
Race/ethnicity (%)		
Native Hawaiian	13.0	14.8
Japanese American	43.2	43.5
White	35.1	30.6
Other	8.7	11.1
Body mass index (kg/m^2^, %) ^b^		
< 22	11.3	29.0
22– < 25	29.1	28.1
25– < 30	43.6	27.1
≥30	15.8	14.6
Education completed (%) ^b^		
high school or less	10.0	10.6
some college	54.2	58.4
college or higher	35.1	30.1
Smoking status (%) ^b^		
Never smoker	30.6	56.8
Past smoker	50.5	27.7
Current smoker	17.1	13.9
Pack-years of cigarette smoking ^a,c^	17.7 ± 15.6	13.9 ± 13.6
History of diabetes (%)	10.3	8.7
History of heart attack (%)	9.3	4.2
History of high blood pressure (%)	38.8	33.1
History of stroke (%)	2.9	1.8
Physical activity >2.5 h/wk (%)	11.3	8.0
Alcohol intake (g/day) ^a^	15.5 ± 31.4	4.5 ± 14.9

^a^ Mean ± standard deviation, ^b^ Percentages may not sum up to 100% due to missing values, ^c^ For current and former smokers only.

**Table 2:** Sepsis-related mortality among non-cancer and cancer patients in the Multiethnic Cohort.

	Any-Cause Deaths					Cancer Deaths ^1,2^				
	*N*	*N*	Sepsis as	Sepsis as	Percent	*N*	*N*	Sepsis	Sepsis as	Percent
Sex/Ethnicity	Participants	Deaths	Primary	Contributing	Sepsis-Rel.	Cancer Cases	Deaths	as Primary	Contributing	Sepsis-Rel.
All participants	103,898	37,386	462	1356	4.86	29,255	16,218	147	619	4.72
Men	48,937	20,722	264	727	4.78	14,992	9194	81	347	4.66
Native Hawaiian	6228	2835	49	116	5.82	1882	1207	15	54	5.72
Japanese American	20,871	8922	108	350	5.13	6719	4174	35	166	4.82
White	17,733	7446	80	189	3.61	5329	3224	25	96	3.75
Other	4105	1519	27	72	6.52	1062	589	6	31	6.28
Women	54,961	16,664	198	629	4.96	14263	7024	66	272	4.81
Native Hawaiian	8118	2875	40	153	6.71	2401	1283	14	62	5.92
Japanese American	23,642	6832	79	269	5.09	6164	2890	29	115	4.98
White	17,310	5518	59	148	3.75	4359	2221	18	65	3.74
Other	5891	1439	20	59	5.49	1339	630	5	30	5.56

^1^ For a number of participants, both cancer and sepsis were coded as contributing COD. ^2^ Numbers exclude deaths with cancer as a COD but no cancer diagnosis.

**Table 3:** Associations of participants’ characteristics with sepsis and other-cause mortality among all participants in the Multiethnic Cohort.

		Men and Women					Men					Women				
		Sepsis	Other-Cause	Relative Risk Ratio			Sepsis	Other-Cause	Relative Risk Ratio			Sepsis	Other-Cause	Relative Risk Ratio		
Characteristic/Level		HR	HR	HR	LCL	UCL	HR	HR	HR	LCL	UCL	HR	HR	HR	LCL	UCL
BMI																
	<22	**1.20**	**1.24**				**1.55**	**1.30**				0.99	**1.21**			
	22–24.9	1.00 (reference)					1.00 (reference)					1.00 (reference)				
	25–29.9	1.04	1.01				1.11	1.00				0.93	1.03			
	30 or higher	**1.93**	**1.30**	**1.48**	**1.03**	**2.11**	**1.87**	**1.32**	**1.87**	**1.15**	**3.04**	**1.94**	**1.23**	1.17	0.69	2.01
Education																
	High school or less	1.00 (reference)					1.00 (reference)					1.00 (reference)				
	Some college	1.03	**0.93**				0.99	**0.92**				1.10	0.95			
	College degree or higher	**0.70**	**0.77**	1.01	0.76	1.35	**0.70**	**0.75**	1.01	0.69	1.48	**0.73**	**0.82**	1.04	0.67	1.60
Tobacco smoking																
	Never smoker	1.00 (reference)					1.00 (reference)					1.00 (reference)				
	Past smoker	1.11	**1.29**				1.13	**1.31**				1.09	**1.29**			
	Current smoker	**2.21**	**2.42**	**0.79**	**0.63**	**0.99**	**2.36**	**2.43**	0.84	0.60	1.17	**2.00**	**2.40**	**0.71**	**0.51**	**0.98**
Alcohol drinking (g/d)																
	None	1.00 (reference)					1.00 (reference)					1.00 (reference)				
	<14	**0.71**	**0.86**				**0.72**	**0.86**				**0.69**	**0.86**			
	14 or more	0.91	**0.93**	0.81	0.65	1.01	0.94	**0.94**	0.84	0.63	1.12	0.77	**0.87**	0.70	0.47	1.05
Physical activity (AC mets/d)																
	<2.5	1.00 (reference)					1.00 (reference)					1.00 (reference)				
	≥2.5	0.88	**0.88**	1.01	0.86	1.17	**0.80**	**0.87**	0.92	0.75	1.12	1.02	**0.87**	1.17	0.92	1.49
History of diabetes																
	No	1.00 (reference)					1.00 (reference)					1.00 (reference)				
	Yes	**2.11**	**1.86**	1.14	0.99	1.30	**2.04**	**1.76**	1.16	0.97	1.39	**2.17**	**2.01**	1.08	0.88	1.31
History of heart disease																
	No	1.00 (reference)					1.00 (reference)					1.00 (reference)				
	Yes	**1.34**	**1.57**	0.86	0.73	1.01	**1.35**	**1.56**	0.86	0.71	1.05	**1.33**	**1.60**	0.84	0.63	1.10
History of hypertension																
	No	1.00 (reference)					1.00 (reference)					1.00 (reference)				
	Yes	**1.31**	**1.26**	1.05	0.94	1.16	**1.20**	**1.25**	0.96	0.83	1.11	**1.45**	**1.24**	1.17	1.00	1.38
History of stroke																
	No	1.00 (reference)					1.00 (reference)					1.00 (reference)				
	Yes	**1.59**	**1.64**	0.97	0.76	1.23	**1.40**	**1.57**	0.89	0.65	1.22	**1.97**	**1.80**	1.09	0.76	1.57
Cancer at any site																
	No	1.00 (reference)					1.00 (reference)					1.00 (reference)				
	Yes	**3.39**	**3.51**	1.01	0.91	1.12	**3.26**	**3.38**	1.00	0.87	1.15	**3.55**	**3.73**	0.99	0.85	1.16

Notes: All baseline factors and cancer status modeled simultaneously in a multivariable model. Bold-face: statistically significant at *p* = 0.05. HR: hazard ratio. LCL: lower confidence limit. UCL: upper confidence limit.

**Table 4:** Associations of participants’ characteristics with sepsis and other-cause mortality among cancer cases in the Multiethnic Cohort.

		Men and Women					Men					Women				
		Sepsis	Other-Cause	Relative Risk Ratio			Sepsis	Other-Cause	Relative Risk Ratio			Sepsis	Other-Cause	Relative Risk Ratio		
Characteristic/Level		HR	HR	HR	LCL	UCL	HR	HR	HR	LCL	UCL	HR	HR	HR	LCL	UCL
BMI																
	<22	0.98	**1.16**				1.02	**1.19**				0.99	**1.14**			
	22–24.9	1.00 (reference)					1.00 (reference)					1.00 (reference)				
	25–29.9	0.98	1.04				0.98	1.02				0.95	1.10			
	30 or higher	**1.43**	**1.22**	0.93	0.52	1.65	1.28	**1.26**	0.85	0.39	1.83	**1.62**	**1.17**	1.04	0.42	2.57
Education																
	High school or less	1.00 (reference)					1.00 (reference)					1.00 (reference)				
	Some college	1.04	**0.92**				0.97	**0.91**				1.17	**0.92**			
	College degree or higher	**0.70**	**0.77**	1.02	0.62	1.69	0.70	**0.76**	0.98	0.51	1.87	0.71	**0.80**	1.14	0.51	2.56
Tobacco smoking																
	Never smoker	1.00 (reference)					1.00 (reference)					1.00 (reference)				
	Past smoker	0.91	**1.35**				1.06	**1.42**				0.75	**1.27**			
	Current smoker	**1.63**	**2.33**	**0.48**	**0.33**	**0.69**	**1.98**	**2.43**	0.61	0.35	1.05	1.33	**2.29**	**0.34**	**0.19**	**0.61**
Alcohol drinking (g/d)																
	None	1.00 (reference)					1.00 (reference)					1.00 (reference)				
	<14	0.83	**0.94**				0.88	0.95				0.79	0.94			
	14 or more	1.04	0.98	0.94	0.66	1.35	1.08	1.00	1.00	0.62	1.60	0.83	0.90	0.77	0.40	1.48
Physical activity (AC mets/d)																
	<2.5	1.00 (reference)					1.00 (reference)					1.00 (reference)				
	≥2.5	1.03	**0.92**	1.12	0.88	1.43	0.88	0.91	0.97	0.71	1.32	1.35	0.94	1.44	0.99	2.11
History of diabetes																
	No	1.00 (reference)					1.00 (reference)					1.00 (reference)				
	Yes	**1.58**	**1.41**	1.12	0.87	1.43	**1.70**	**1.41**	1.21	0.88	1.67	1.37	**1.40**	0.98	0.66	1.46
History of heart disease																
	No	1.00 (reference)					1.00 (reference)					1.00 (reference)				
	Yes	1.26	**1.25**	1.00	0.75	1.35	1.35	**1.30**	1.03	0.73	1.46	1.03	**1.15**	0.90	0.51	1.58
History of hypertension																
	No	1.00 (reference)					1.00 (reference)					1.00 (reference)				
	Yes	0.99	**1.11**	0.89	0.75	1.07	0.85	**1.11**	**0.77**	**0.61**	**0.97**	1.22	**1.11**	1.10	0.84	1.46
History of stroke																
	No	1.00 (reference)					1.00 (reference)					1.00 (reference)				
	Yes	1.40	**1.36**	1.03	0.64	1.66	1.49	**1.23**	1.21	0.70	2.10	1.14	**1.77**	0.64	0.23	1.77
Colorectal cancer																
	No	1.00 (reference)					1.00 (reference)					1.00 (reference)				
	Yes	1.06	**0.79**	**1.40**	**1.14**	**1.72**	1.07	**0.76**	**1.43**	**1.10**	**1.86**	1.00	**0.81**	1.33	0.95	1.87
Lung cancer																
	No	1.00 (reference)					1.00 (reference)					1.00 (reference)				
	Yes	1.22	**3.00**	**0.39**	**0.29**	**0.53**	**1.42**	**3.08**	**0.44**	**0.31**	**0.63**	0.86	**2.87**	**0.27**	**0.15**	**0.48**
Skin cancer																
	No	1.00 (reference)					1.00 (reference)					1.00 (reference)				
	Yes	**0.59**	**0.80**	0.68	0.39	1.16	**0.50**	**0.80**	0.64	0.33	1.26	0.76	**0.79**	0.77	0.31	1.90
Breast cancer																
	No	1.00 (reference)					1.00 (reference)					1.00 (reference)				
	Yes	**0.54**	**0.60**	0.89	0.70	1.13	N/A	N/A				**0.55**	**0.61**	0.90	0.69	1.17
Prostate cancer																
	No	1.00 (reference)					1.00 (reference)					1.00 (reference)				
	Yes	**0.55**	**0.54**	1.00	0.80	1.25	**0.52**	**0.52**	0.97	0.76	1.22	N/A	N/A			
Chemotherapy																
	No	1.00 (reference)					1.00 (reference)					1.00 (reference)				
	Yes	**1.31**	**1.21**	1.12	0.94	1.32	**1.52**	**1.39**	1.12	0.89	1.42	1.12	1.03	1.14	0.89	1.47
Radiation																
	No	1.00 (reference)					1.00 (reference)					1.00 (reference)				
	Yes	0.90	**1.16**	**0.76**	**0.64**	**0.91**	0.95	**1.20**	0.81	0.65	1.01	0.82	**1.12**	**0.70**	**0.53**	**0.92**

Notes: All baseline factors modeled simultaneously in a multivariable model. Cancer status and treatment analyses adjusted for all baseline factors. Bold-face: statistically significant at *p* = 0.05. HR: hazard ratio. LCL: lower confidence limit. UCL: upper confidence limit.

## Data Availability

The data presented in this study are available on reasonable request from the Multiethnic Cohort. The data are not publicly available because they contain protected health information.

## Supplementary Materials

The following are available online at https://www.mdpi.com/2075-4426/11/2/146/s1, Table S1: Associations of participants’ characteristics with sepsis and all-cause mortality among all participants in the Multiethnic Cohort, by sex and race/ethnicity., Table S2: Associations of participants’ characteristics with sepsis and all-cause mortality among cancer cases in the Multiethnic Cohort, by sex and race/ethnicity.
