# Supplementary material for: Association of Sepsis Mortality with Specific Cancer Sites and Treatment Type: The Multiethnic Cohort Study
Source: J Pers Med. 2021 Feb 19;11(2):146. doi: 10.3390/jpm11020146 (PMC7922684; doi:10.3390/jpm11020146)
Supplement: Supplementary file 1 [file jpm-11-00146-s001.pdf]

Supplementary Table S1. Associations of participants' characteristics with sepsis and all-cause mortality among all participants in the Multiethnic Cohort, by sex and race/ethnicity.

		Men and women					Men					Women				
Race/		Sepsis	Other-cause	Relative risk ratio			Sepsis	Other-cause	Relative risk ratio			Sepsis	Other-cause	Relative risk ratio		
Ethnicity	Characteristic / Level	HR	HR	HR	LCL	UCL	HR	HR	HR	LCL	UCL	HR	HR	HR	LCL	UCL
All races/ethnicities																
	BMI															
	< 22	**1.20**	**1.24**				**1.55**	**1.30**				0.99	**1.21**			
	22 - 24.9	1.00	(reference)				1.00	(reference)				1.00	(reference)			
	25 - 29.9	1.04	1.01				1.11	1.00				0.93	1.03			
	30 or higher	**1.93**	**1.30**	**1.48**	**1.03**	**2.11**	**1.87**	**1.32**	**1.87**	**1.15**	**3.04**	**1.94**	**1.23**	1.17	0.69	2.01
	Education															
	High school or less	1.00	(reference)				1.00	(reference)				1.00	(reference)			
	Some college	1.03	**0.93**				0.99	**0.92**				1.10	**0.95**			
	College degree or higher	**0.70**	**0.77**	1.01	0.76	1.35	**0.70**	**0.75**	1.01	0.69	1.48	**0.73**	**0.82**	1.04	0.67	1.60
	Tobacco smoking															
	Never smoker	1.00	(reference)				1.00	(reference)				1.00	(reference)			
	Past smoker	1.11	**1.29**				1.13	**1.31**				1.09	**1.29**			
	Current smoker	**2.21**	**2.42**	**0.79**	**0.63**	**0.99**	**2.36**	**2.43**	0.84	0.60	1.17	**2.00**	**2.40**	**0.71**	**0.51**	**0.98**
	Alcohol drinking (g/d)															
	None	1.00	(reference)				1.00	(reference)				1.00	(reference)			
	< 14	**0.71**	**0.86**				**0.72**	**0.86**				**0.69**	**0.86**			
	14 or more	0.91	**0.93**	0.81	0.65	1.01	0.94	**0.94**	0.84	0.63	1.12	0.77	**0.87**	0.70	0.47	1.05
	Physical activity (AC mets/d)															
	< 2.5	1.00	(reference)				1.00	(reference)				1.00	(reference)			
	≥ 2.5	0.88	**0.88**	1.01	0.86	1.17	**0.80**	**0.87**	0.92	0.75	1.12	1.02	**0.87**	1.17	0.92	1.49
	History of diabetes															
	No	1.00	(reference)				1.00	(reference)				1.00	(reference)			
	Yes	**2.11**	**1.86**	1.14	0.99	1.30	**2.04**	**1.76**	1.16	0.97	1.39	**2.17**	**2.01**	1.08	0.88	1.31
	History of heart disease															
	No	1.00	(reference)				1.00	(reference)				1.00	(reference)			
	Yes	**1.34**	**1.57**	0.86	0.73	1.01	**1.35**	**1.56**	0.86	0.71	1.05	**1.33**	**1.60**	0.84	0.63	1.10
	History of hypertension															
	No	1.00	(reference)				1.00	(reference)				1.00	(reference)			
	Yes	**1.31**	**1.26**	1.05	0.94	1.16	**1.20**	**1.25**	0.96	0.83	1.11	**1.45**	**1.24**	1.17	1.00	1.38
	History of stroke															
	No	1.00	(reference)				1.00	(reference)				1.00	(reference)			
	Yes	**1.59**	**1.64**	0.97	0.76	1.23	**1.40**	**1.57**	0.89	0.65	1.22	**1.97**	**1.80**	1.09	0.76	1.57
	Cancer at any site															
	No	1.00	(reference)				1.00	(reference)				1.00	(reference)			
	Yes	**3.39**	**3.51**	1.01	0.91	1.12	**3.26**	**3.38**	1.00	0.87	1.15	**3.55**	**3.73**	0.99	0.85	1.16
Native Hawaiians																
	BMI															
	< 22	0.90	**1.16**				1.86	**1.29**				0.52	1.11			
	22 - 24.9	1.00	(reference)				1.00	(reference)				1.00	(reference)			
	25 - 29.9	1.05	**0.88**				1.16	**0.82**				1.00	0.95			
	30 or higher	**2.16**	**1.14**	1.75	0.57	5.39	**2.50**	1.08	4.71	0.86	25.76	**1.90**	**1.16**	0.80	0.17	3.69
	Education															
	High school or less	1.00	(reference)				1.00	(reference)				1.00	(reference)			
	Some college	1.12	**0.82**				1.05	**0.84**				1.19	**0.81**			
	College degree or higher	0.75	**0.68**	1.51	0.76	3.00	0.78	**0.67**	1.47	0.58	3.73	0.72	**0.70**	1.51	0.55	4.14
	Tobacco smoking															
	Never smoker	1.00	(reference)				1.00	(reference)				1.00	(reference)			
	Past smoker	0.85	**1.32**				0.87	**1.28**				0.88	**1.35**			
	Current smoker	**1.78**	**2.36**	**0.49**	**0.30**	**0.80**	**2.29**	**2.29**	0.68	0.32	1.46	1.46	**2.45**	**0.39**	**0.20**	**0.76**
	Alcohol drinking (g/d)															
	None	1.00	(reference)				1.00	(reference)				1.00	(reference)			
	< 14	0.92	**0.86**				1.24	**0.85**				0.66	**0.88**			
	14 or more	0.83	**0.90**	0.98	0.59	1.64	0.90	**0.90**	1.47	0.72	3.00	0.88	0.93	0.70	0.29	1.72
	Physical activity (AC mets/d)															
	< 2.5	1.00	(reference)				1.00	(reference)				1.00	(reference)			
	≥ 2.5	**0.68**	**0.81**	0.84	0.58	1.20	0.67	**0.82**	0.82	0.51	1.32	0.71	**0.79**	0.90	0.51	1.60
	History of diabetes															
	No	1.00	(reference)				1.00	(reference)				1.00	(reference)			
	Yes	**2.29**	**2.02**	1.13	0.87	1.48	**2.31**	**1.90**	1.21	0.83	1.78	**2.25**	**2.20**	1.02	0.71	1.48
	History of heart disease															
	No	1.00	(reference)				1.00	(reference)				1.00	(reference)			
	Yes	**1.81**	**1.63**	1.11	0.80	1.53	**1.92**	**1.66**	1.15	0.73	1.81	**1.78**	**1.60**	1.12	0.70	1.78
	History of hypertension															
	No	1.00	(reference)				1.00	(reference)				1.00	(reference)			

Yes	1.15	**1.27**	0.90	0.70	1.15	1.02	**1.20**	0.86	0.60	1.22	1.31	**1.36**	0.96	0.68	1.36
History of stroke															
No	1.00	(reference)				1.00	(reference)				1.00	(reference)			
Yes	1.39	**1.56**	0.89	0.52	1.52	0.67	**1.47**	0.45	0.16	1.28	**2.18**	**1.74**	1.26	0.67	2.37
Cancer at any site															
No	1.00	(reference)				1.00	(reference)				1.00	(reference)			
Yes	**3.48**	**4.08**	0.85	0.67	1.08	**3.39**	**4.11**	0.79	0.55	1.11	**3.39**	**4.19**	0.83	0.60	1.16
Japanese Americans															
BMI															
< 22	1.13	**1.28**				**1.43**	**1.39**				0.92	**1.18**			
22 - 24.9	1.00	(reference)				1.00	(reference)				1.00	(reference)			
25 - 29.9	1.06	**1.05**				1.12	**1.07**				0.95	1.01			
30 or higher	**1.80**	**1.40**	1.15	0.68	1.93	**1.65**	**1.45**	1.23	0.62	2.45	**1.83**	**1.27**	1.05	0.47	2.33
Education															
High school or less	1.00	(reference)				1.00	(reference)				1.00	(reference)			
Some college	1.05	**0.91**				0.98	**0.89**				1.20	0.95			
College degree or higher	0.81	**0.75**	1.25	0.81	1.92	0.79	**0.72**	1.20	0.67	2.15	0.88	**0.80**	1.40	0.73	2.69
Tobacco smoking															
Never smoker	1.00	(reference)				1.00	(reference)				1.00	(reference)			
Past smoker	**1.20**	**1.24**				**1.29**	**1.27**				1.07	**1.25**			
Current smoker	**2.06**	**2.10**	0.95	0.67	1.34	**2.18**	**2.13**	1.04	0.63	1.72	**1.90**	**2.08**	0.78	0.45	1.37
Alcohol drinking (g/d)															
None	1.00	(reference)				1.00	(reference)				1.00	(reference)			
< 14	**0.71**	**0.85**				**0.62**	**0.86**				0.92	**0.84**			
14 or more	0.99	**0.91**	0.91	0.65	1.29	0.92	**0.91**	0.73	0.48	1.11	1.12	0.90	1.35	0.53	3.49
Physical activity (AC mets/d)															
< 2.5	1.00	(reference)				1.00	(reference)				1.00	(reference)			
≥ 2.5	0.89	**0.88**	1.01	0.79	1.30	0.79	**0.89**	0.89	0.65	1.22	1.10	**0.86**	1.27	0.85	1.90
History of diabetes															
No	1.00	(reference)				1.00	(reference)				1.00	(reference)			
Yes	**1.78**	**1.68**	1.06	0.87	1.29	**1.64**	**1.59**	1.03	0.79	1.33	**2.00**	**1.82**	1.10	0.81	1.49
History of heart disease															
No	1.00	(reference)				1.00	(reference)				1.00	(reference)			
Yes	1.20	**1.61**	**0.75**	**0.57**	**0.97**	1.25	**1.61**	0.78	0.57	1.06	1.01	**1.59**	0.63	0.37	1.08
History of hypertension															
No	1.00	(reference)				1.00	(reference)				1.00	(reference)			
Yes	**1.33**	**1.18**	1.13	0.96	1.32	**1.28**	**1.18**	1.08	0.88	1.33	**1.41**	**1.16**	1.22	0.95	1.55
History of stroke															
No	1.00	(reference)				1.00	(reference)				1.00	(reference)			
Yes	**1.55**	**1.73**	0.89	0.62	1.29	1.31	**1.64**	0.80	0.51	1.26	**2.11**	**1.96**	1.07	0.59	1.96
Cancer at any site															
No	1.00	(reference)				1.00	(reference)				1.00	(reference)			
Yes	**3.14**	**3.46**	0.95	0.81	1.11	**3.02**	**3.40**	0.91	0.74	1.12	**3.35**	**3.61**	1.00	0.78	1.26
Whites															
BMI															
< 22	**1.43**	**1.22**				**1.87**	**1.25**				1.23	**1.23**			
22 - 24.9	1.00	(reference)				1.00	(reference)				1.00	(reference)			
25 - 29.9	1.09	1.02				1.16	0.98				1.02	**1.10**			
30 or higher	**2.04**	**1.30**	1.97	0.94	4.14	**1.90**	**1.32**	2.56	0.95	6.90	**2.15**	**1.25**	1.61	0.52	4.98
Education															
High school or less	1.00	(reference)				1.00	(reference)				1.00	(reference)			
Some college	1.12	1.04				1.42	1.00				0.94	**1.13**			
College degree or higher	0.80	**0.87**	0.99	0.48	2.02	1.04	**0.81**	1.80	0.65	5.04	0.64	1.00	0.53	0.19	1.51
Tobacco smoking															
Never smoker	1.00	(reference)				1.00	(reference)				1.00	(reference)			
Past smoker	1.25	**1.39**				1.19	**1.40**				**1.44**	**1.40**			
Current smoker	**2.85**	**2.96**	0.86	0.54	1.38	**3.17**	**3.15**	0.85	0.44	1.66	**2.61**	**2.73**	0.98	0.50	1.93
Alcohol drinking (g/d)															
None	1.00	(reference)				1.00	(reference)				1.00	(reference)			
< 14	**0.66**	**0.85**				**0.65**	**0.86**				0.70	**0.85**			
14 or more	0.97	**0.92**	0.81	0.52	1.26	1.09	0.97	0.86	0.46	1.58	0.84	**0.84**	0.83	0.42	1.63
Physical activity (AC mets/d)															
< 2.5	1.00	(reference)				1.00	(reference)				1.00	(reference)			
≥ 2.5	1.12	**0.94**	1.20	0.92	1.56	1.09	**0.92**	1.18	0.84	1.65	1.20	0.95	1.26	0.84	1.90
History of diabetes															
No	1.00	(reference)				1.00	(reference)				1.00	(reference)			
Yes	**2.70**	**1.98**	1.37	1.00	1.87	**2.68**	**1.95**	1.38	0.90	2.10	**2.81**	**2.05**	1.37	0.85	2.20
History of heart disease															
No	1.00	(reference)				1.00	(reference)				1.00	(reference)			
Yes	**1.44**	**1.49**	0.97	0.71	1.32	**1.51**	**1.47**	1.03	0.71	1.50	1.35	**1.57**	0.86	0.50	1.49

History of hypertension															
No	1.00	(reference)				1.00	(reference)				1.00	(reference)			
Yes	1.16	**1.35**	0.86	0.69	1.07	0.99	**1.36**	**0.73**	**0.54**	**0.97**	**1.41**	**1.31**	1.08	0.77	1.50
History of stroke															
No	1.00	(reference)				1.00	(reference)				1.00	(reference)			
Yes	1.29	**1.54**	0.84	0.49	1.44	1.66	**1.47**	1.13	0.60	2.14	0.93	**1.70**	0.55	0.20	1.52
Cancer at any site															
No	1.00	(reference)				1.00	(reference)				1.00	(reference)			
Yes	**3.46**	**3.20**	1.14	0.93	1.39	**3.57**	**3.09**	1.24	0.95	1.62	**3.43**	**3.39**	1.02	0.75	1.38

Notes: 1. All baseline factors and cancer status modeled simultaneously in a multivariable model.
2. Bold-face: statistically significant at α=0.05. HR: hazard ratio. LCL: lower confidence limit. UCL: upper confidence limit.

Supplementary Table S2. Associations of participants' characteristics with sepsis and all-cause mortality among cancer cases in the Multiethnic Cohort, by sex and race/ethnicity.

		Men and women					Men					Women				
Race/		Sepsis	Other-cause	Relative risk ratio			Sepsis	Other-cause	Relative risk ratio			Sepsis	Other-cause	Relative risk ratio		
Ethnicity	Characteristic / Level	HR	HR	HR	LCL	UCL	HR	HR	HR	LCL	UCL	HR	HR	HR	LCL	UCL
All races/ethnicities																
	BMI															
	< 22	0.98	**1.16**				1.02	**1.19**				0.99	**1.14**			
	22 - 24.9	1.00	(reference)				1.00	(reference)				1.00	(reference)			
	25 - 29.9	0.98	1.04				0.98	1.02				0.95	**1.10**			
	30 or higher	**1.43**	**1.22**	0.93	0.52	1.65	1.28	**1.26**	0.85	0.39	1.83	**1.62**	**1.17**	1.04	0.42	2.57
	Education															
	High school or less	1.00	(reference)				1.00	(reference)				1.00	(reference)			
	Some college	1.04	**0.92**				0.97	**0.91**				1.17	**0.92**			
	College degree or higher	**0.70**	**0.77**	1.02	0.62	1.69	0.70	**0.76**	0.98	0.51	1.87	0.71	**0.80**	1.14	0.51	2.56
	Tobacco smoking															
	Never smoker	1.00	(reference)				1.00	(reference)				1.00	(reference)			
	Past smoker	0.91	**1.35**				1.06	**1.42**				0.75	**1.27**			
	Current smoker	**1.63**	**2.33**	**0.48**	**0.33**	**0.69**	**1.98**	**2.43**	0.61	0.35	1.05	1.33	**2.29**	**0.34**	**0.19**	**0.61**
	Alcohol drinking (g/d)															
	None	1.00	(reference)				1.00	(reference)				1.00	(reference)			
	< 14	0.83	**0.94**				0.88	0.95				0.79	0.94			
	14 or more	1.04	0.98	0.94	0.66	1.35	1.08	1.00	1.00	0.62	1.60	0.83	0.90	0.77	0.40	1.48
	Physical activity (AC mets/d)															
	< 2.5	1.00	(reference)				1.00	(reference)				1.00	(reference)			
	≥ 2.5	1.03	**0.92**	1.12	0.88	1.43	0.88	**0.91**	0.97	0.71	1.32	1.35	0.94	1.44	0.99	2.11
	History of diabetes															
	No	1.00	(reference)				1.00	(reference)				1.00	(reference)			
	Yes	**1.58**	**1.41**	1.12	0.87	1.43	**1.70**	**1.41**	1.21	0.88	1.67	1.37	**1.40**	0.98	0.66	1.46
	History of heart disease															
	No	1.00	(reference)				1.00	(reference)				1.00	(reference)			
	Yes	1.26	**1.25**	1.00	0.75	1.35	1.35	**1.30**	1.03	0.73	1.46	1.03	**1.15**	0.90	0.51	1.58
	History of hypertension															
	No	1.00	(reference)				1.00	(reference)				1.00	(reference)			
	Yes	0.99	**1.11**	0.89	0.75	1.07	0.85	**1.11**	**0.77**	**0.61**	**0.97**	1.22	**1.11**	1.10	0.84	1.46
	History of stroke															
	No	1.00	(reference)				1.00	(reference)				1.00	(reference)			
	Yes	1.40	**1.36**	1.03	0.64	1.66	1.49	**1.23**	1.21	0.70	2.10	1.14	**1.77**	0.64	0.23	1.77
	Colorectal cancer															
	No	1.00	(reference)				1.00	(reference)				1.00	(reference)			
	Yes	1.06	**0.79**	**1.40**	**1.14**	**1.72**	1.07	**0.76**	**1.43**	**1.10**	**1.86**	1.00	**0.81**	1.33	0.95	1.87
	Lung cancer															
	No	1.00	(reference)				1.00	(reference)				1.00	(reference)			
	Yes	1.22	**3.00**	**0.39**	**0.29**	**0.53**	**1.42**	**3.08**	**0.44**	**0.31**	**0.63**	0.86	**2.87**	**0.27**	**0.15**	**0.48**
	Skin cancer															
	No	1.00	(reference)				1.00	(reference)				1.00	(reference)			
	Yes	**0.59**	**0.80**	0.68	0.39	1.16	**0.50**	**0.80**	0.64	0.33	1.26	0.76	**0.79**	0.77	0.31	1.90
	Breast cancer															
	No	1.00	(reference)				1.00	(reference)				1.00	(reference)			
	Yes	**0.54**	**0.60**	0.89	0.70	1.13	N/A	N/A				**0.55**	**0.61**	0.90	0.69	1.17
	Prostate cancer															
	No	1.00	(reference)				1.00	(reference)				1.00	(reference)			
	Yes	**0.55**	**0.54**	1.00	0.80	1.25	**0.52**	**0.52**	0.97	0.76	1.22	N/A	N/A			
	Chemotherapy															
	No	1.00	(reference)				1.00	(reference)				1.00	(reference)			
	Yes	**1.31**	**1.21**	1.12	0.94	1.32	**1.52**	**1.39**	1.12	0.89	1.42	1.12	1.03	1.14	0.89	1.47
	Radiation															
	No	1.00	(reference)				1.00	(reference)				1.00	(reference)			
	Yes	0.90	**1.16**	**0.76**	**0.64**	**0.91**	0.95	**1.20**	0.81	0.65	1.01	0.82	**1.12**	**0.70**	**0.53**	**0.92**
Native Hawaiians																
	BMI															
	< 22	0.93	1.02				2.21	1.11				0.39	0.97			
	22 - 24.9	1.00	(reference)				1.00	(reference)				1.00	(reference)			
	25 - 29.9	1.00	**0.80**				0.93	**0.70**				1.17	0.94			
	30 or higher	1.62	1.01	1.82	0.31	10.73	1.53	0.95	4.22	0.36	49.27	1.70	1.06	0.80	0.05	11.59
	Education															
	High school or less	1.00	(reference)				1.00	(reference)				1.00	(reference)			
	Some college	0.70	**0.83**				0.56	0.89				1.03	**0.78**			
	College degree or higher	0.59	**0.72**	0.68	0.23	2.03	0.43	0.83	0.32	0.08	1.40	1.06	**0.61**	2.28	0.38	13.75
	Tobacco smoking															

Never smoker	1.00	(reference)				1.00	(reference)				1.00	(reference)			
Past smoker	0.70	**1.35**				1.48	**1.47**				**0.34**	1.18			
Current smoker	1.02	**2.13**	**0.25**	**0.11**	**0.57**	1.95	**2.16**	0.90	0.22	3.64	0.63	**2.24**	**0.08**	**0.02**	**0.27**
Alcohol drinking (g/d)															
None	1.00	(reference)				1.00	(reference)				1.00	(reference)			
< 14	1.39	1.04				**2.39**	1.18				0.86	1.02			
14 or more	0.71	0.99	0.96	0.40	2.32	1.05	1.11	1.93	0.55	6.75	0.31	1.00	0.27	0.03	2.45
Physical activity (AC mets/d)															
< 2.5	1.00	(reference)				1.00	(reference)				1.00	(reference)			
≥ 2.5	0.99	**0.81**	1.22	0.70	2.11	0.69	**0.70**	0.98	0.46	2.10	1.69	1.01	1.68	0.75	3.74
History of diabetes															
No	1.00	(reference)				1.00	(reference)				1.00	(reference)			
Yes	1.59	**1.53**	1.04	0.62	1.73	**2.24**	**1.53**	1.47	0.73	2.98	1.31	**1.66**	0.79	0.37	1.71
History of heart disease															
No	1.00	(reference)				1.00	(reference)				1.00	(reference)			
Yes	1.45	**1.23**	1.18	0.62	2.25	1.36	**1.43**	0.95	0.41	2.21	1.55	1.00	1.54	0.56	4.25
History of hypertension															
No	1.00	(reference)				1.00	(reference)				1.00	(reference)			
Yes	0.86	**1.13**	0.76	0.50	1.15	0.79	0.96	0.82	0.45	1.50	0.91	**1.33**	0.68	0.38	1.23
History of stroke															
No	1.00	(reference)				1.00	(reference)				1.00	(reference)			
Yes	1.63	**1.34**	1.21	0.41	3.53	1.16	1.15	1.00	0.22	4.58	2.93	**1.92**	1.53	0.33	6.96
Colorectal cancer															
No	1.00	(reference)				1.00	(reference)				1.00	(reference)			
Yes	1.11	**0.77**	1.45	0.86	2.45	1.10	**0.83**	1.45	0.70	3.00	1.14	**0.70**	1.56	0.70	3.47
Lung cancer															
No	1.00	(reference)				1.00	(reference)				1.00	(reference)			
Yes	1.44	**3.16**	**0.38**	**0.20**	**0.74**	1.25	**2.78**	**0.35**	**0.15**	**0.83**	1.46	**3.64**	0.37	0.13	1.08
Skin cancer															
No	1.00	(reference)				1.00	(reference)				1.00	(reference)			
Yes	0.50	**0.62**	0.81	0.11	6.07	0.83	**0.51**	1.66	0.21	12.95	0.00	0.99	0.00	0.00	inf
Breast cancer															
No	1.00	(reference)				1.00	(reference)				1.00	(reference)			
Yes	0.64	**0.61**	1.12	0.70	1.79	N/A	N/A				0.64	**0.61**	1.17	0.69	1.98
Prostate cancer															
No	1.00	(reference)				1.00	(reference)				1.00	(reference)			
Yes	0.64	**0.49**	1.49	0.89	2.51	**0.55**	**0.46**	1.36	0.76	2.44	N/A	N/A			
Chemotherapy															
No	1.00	(reference)				1.00	(reference)				1.00	(reference)			
Yes	1.21	**1.12**	1.03	0.70	1.53	1.13	**1.24**	0.93	0.50	1.74	1.29	1.09	1.11	0.65	1.89
Radiation															
No	1.00	(reference)				1.00	(reference)				1.00	(reference)			
Yes	0.81	1.02	0.70	0.46	1.04	0.89	1.08	0.81	0.46	1.42	0.67	0.98	**0.52**	**0.28**	**0.94**
Japanese Americans															
BMI															
< 22	0.83	**1.15**				0.87	**1.26**				0.78	1.02			
22 - 24.9	1.00	(reference)				1.00	(reference)				1.00	(reference)			
25 - 29.9	0.99	**1.08**				1.00	**1.10**				0.96	1.08			
30 or higher	0.93	**1.30**	0.47	0.20	1.13	0.95	**1.37**	0.44	0.14	1.41	0.82	1.19	0.47	0.12	1.85
Education															
High school or less	1.00	(reference)				1.00	(reference)				1.00	(reference)			
Some college	1.14	**0.89**				1.07	**0.86**				1.24	0.91			
College degree or higher	0.94	**0.79**	1.53	0.70	3.34	0.99	**0.74**	1.67	0.60	4.68	0.86	0.89	1.33	0.39	4.50
Tobacco smoking															
Never smoker	1.00	(reference)				1.00	(reference)				1.00	(reference)			
Past smoker	0.91	**1.29**				0.97	**1.29**				0.85	**1.33**			
Current smoker	**1.60**	**2.08**	**0.54**	**0.31**	**0.95**	**1.74**	**2.12**	0.62	0.28	1.39	1.35	**2.05**	0.42	0.16	1.09
Alcohol drinking (g/d)															
None	1.00	(reference)				1.00	(reference)				1.00	(reference)			
< 14	0.80	**0.91**				0.76	**0.91**				0.89	0.91			
14 or more	1.17	1.03	1.00	0.58	1.73	1.08	1.04	0.87	0.44	1.70	1.96	0.96	1.98	0.54	7.22
Physical activity (AC mets/d)															
< 2.5	1.00	(reference)				1.00	(reference)				1.00	(reference)			
≥ 2.5	1.09	0.93	1.18	0.81	1.72	1.01	0.94	1.07	0.67	1.71	1.28	0.90	1.42	0.76	2.67
History of diabetes															
No	1.00	(reference)				1.00	(reference)				1.00	(reference)			
Yes	**1.76**	**1.29**	1.36	0.97	1.91	**1.85**	**1.31**	1.41	0.92	2.16	1.58	**1.23**	1.28	0.73	2.24
History of heart disease															
No	1.00	(reference)				1.00	(reference)				1.00	(reference)			
Yes	1.19	**1.41**	0.85	0.52	1.38	1.27	**1.47**	0.86	0.49	1.51	0.96	1.21	0.80	0.28	2.24

History of hypertension															
No	1.00	(reference)				1.00	(reference)				1.00	(reference)			
Yes	0.90	**1.07**	0.84	0.65	1.09	0.75	1.07	**0.70**	**0.50**	**0.98**	1.22	1.07	1.13	0.74	1.72
History of stroke															
No	1.00	(reference)				1.00	(reference)				1.00	(reference)			
Yes	1.21	**1.34**	0.90	0.41	1.97	1.08	**1.27**	0.85	0.34	2.15	1.77	**1.79**	0.99	0.23	4.27
Colorectal cancer															
No	1.00	(reference)				1.00	(reference)				1.00	(reference)			
Yes	1.07	**0.74**	**1.48**	**1.11**	**1.96**	1.12	**0.69**	**1.57**	**1.10**	**2.24**	0.91	**0.81**	1.26	0.78	2.04
Lung cancer															
No	1.00	(reference)				1.00	(reference)				1.00	(reference)			
Yes	1.50	**3.03**	**0.50**	**0.32**	**0.77**	**1.82**	**3.11**	**0.60**	**0.37**	**0.98**	0.75	**2.90**	**0.21**	**0.07**	**0.69**
Skin cancer															
No	1.00	(reference)				1.00	(reference)				1.00	(reference)			
Yes	0.96	0.90	1.19	0.37	3.86	0.58	0.69	0.95	0.13	7.13	1.40	1.22	1.30	0.30	5.58
Breast cancer															
No	1.00	(reference)				1.00	(reference)				1.00	(reference)			
Yes	**0.44**	**0.56**	0.79	0.54	1.17	N/A	N/A				**0.42**	**0.56**	0.75	0.50	1.14
Prostate cancer															
No	1.00	(reference)				1.00	(reference)				1.00	(reference)			
Yes	**0.42**	**0.49**	0.84	0.59	1.17	**0.40**	**0.47**	0.81	0.56	1.16	N/A	N/A			
Chemotherapy															
No	1.00	(reference)				1.00	(reference)				1.00	(reference)			
Yes	**1.34**	**1.25**	1.09	0.85	1.41	**1.53**	**1.45**	1.06	0.76	1.50	1.11	1.02	1.15	0.78	1.69
Radiation															
No	1.00	(reference)				1.00	(reference)				1.00	(reference)			
Yes	0.89	**1.18**	**0.75**	**0.58**	**0.97**	0.96	**1.20**	0.80	0.58	1.11	0.82	**1.14**	0.68	0.44	1.05
Whites															
BMI															
< 22	**7.45**	**1.23**				0.97	1.16				1.19	1.12			
22 - 24.9	1.00	(reference)				1.00	(reference)				1.00	(reference)			
25 - 29.9	**6.16**	**1.12**				1.06	1.04				0.74	1.11			
30 or higher	0.74	**1.30**	**19.10**	**1.17**	**312.0**	1.03	**1.27**	0.70	0.15	3.33	2.08	1.11	1.32	0.20	8.90
Education															
High school or less	1.00	(reference)				1.00	(reference)				1.00	(reference)			
Some college	**0.00**	0.94				1.88	0.97				1.22	1.06			
College degree or higher	**0.07**	**0.76**	**0.00**	**0.00**	**0.002**	1.36	**0.80**	3.28	0.41	25.91	0.71	0.87	0.95	0.08	11.06
Tobacco smoking															
Never smoker	1.00	(reference)				1.00	(reference)				1.00	(reference)			
Past smoker	**3.96**	**1.51**				1.21	**1.57**				0.91	1.13			
Current smoker	0.24	**3.09**	0.21	0.01	3.36	**2.71**	**3.39**	0.61	0.20	1.87	1.49	**2.00**	0.60	0.18	1.99
Alcohol drinking (g/d)															
None	1.00	(reference)				1.00	(reference)				1.00	(reference)			
< 14	0.61	**0.90**				0.53	0.92				0.91	0.93			
14 or more	0.77	**0.90**	0.58	0.20	1.67	1.04	0.93	0.65	0.23	1.83	0.98	0.93	1.03	0.30	3.57
Physical activity (AC mets/d)															
< 2.5	1.00	(reference)				1.00	(reference)				1.00	(reference)			
≥ 2.5	0.11	1.02	0.11	0.01	1.66	1.03	1.03	1.00	0.57	1.76	1.13	1.00	1.13	0.54	2.37
History of diabetes															
No	1.00	(reference)				1.00	(reference)				1.00	(reference)			
Yes	**13.63**	**1.58**	**8.60**	**4.59**	**16.11**	0.43	**1.54**	0.28	0.07	1.19	1.11	**1.79**	0.62	0.17	2.25
History of heart disease															
No	1.00	(reference)				1.00	(reference)				1.00	(reference)			
Yes	0.03	**1.18**	0.02	0.00	2.37	1.29	**1.16**	1.11	0.57	2.17	2.03	**1.42**	1.43	0.49	4.11
History of hypertension															
No	1.00	(reference)				1.00	(reference)				1.00	(reference)			
Yes	**0.21**	**1.20**	**0.17**	**0.09**	**0.33**	0.77	**1.26**	0.61	0.37	1.02	1.19	1.08	1.11	0.60	2.05
History of stroke															
No	1.00	(reference)				1.00	(reference)				1.00	(reference)			
Yes	inf	**1.32**	inf	0.00		**3.28**	1.10	**2.97**	**1.16**	**7.65**	0.43	**2.03**	0.21	0.01	3.55
Colorectal cancer															
No	1.00	(reference)				1.00	(reference)				1.00	(reference)			
Yes	1.11	**0.88**	1.30	0.84	2.00	1.22	**0.88**	1.44	0.85	2.46	0.91	0.87	1.05	0.49	2.27
Lung cancer															
No	1.00	(reference)				1.00	(reference)				1.00	(reference)			
Yes	0.74	**2.97**	**0.24**	**0.12**	**0.48**	0.73	**3.28**	**0.20**	**0.08**	**0.49**	0.69	**2.59**	**0.29**	**0.10**	**0.84**
Skin cancer															
No	1.00	(reference)				1.00	(reference)				1.00	(reference)			
Yes	0.62	**0.76**	0.71	0.37	1.36	0.54	**0.80**	0.69	0.32	1.52	0.72	**0.67**	0.76	0.24	2.49
Breast cancer															

No	1.00	(reference)				1.00	(reference)				1.00	(reference)			
Yes	0.65	**0.67**	0.96	0.60	1.51	N/A	N/A				0.66	**0.68**	0.96	0.58	1.60
Prostate cancer															
No	1.00	(reference)				1.00	(reference)				1.00	(reference)			
Yes	**0.68**	**0.63**	1.12	0.77	1.64	0.69	**0.61**	1.17	0.78	1.75	N/A	N/A			
Chemotherapy															
No	1.00	(reference)				1.00	(reference)				1.00	(reference)			
Yes	1.35	**1.24**	1.14	0.80	1.61	1.52	**1.44**	1.09	0.69	1.73	1.15	1.00	1.24	0.73	2.13
Radiation															
No	1.00	(reference)				1.00	(reference)				1.00	(reference)			
Yes	0.93	**1.20**	0.82	0.59	1.14	1.03	**1.23**	0.85	0.57	1.28	0.84	**1.19**	0.77	0.44	1.32

Notes: 1. All baseline factors modeled simultaneously in a multivariable model.
2. Cancer status and treatment analyses adjusted for all baseline factors.
3. Bold-face: statistically significant at α=0.05. HR: hazard ratio. LCL: lower confidence limit. UCL: upper confidence limit.
